# Letter Addressed to the Medical Profession Generally, Relative to Vaccination

**Published:** 1821-04

**Authors:** Edward Jenner


### *Letter addressed to the Medical Profession generally, relative to Vaccination.* By Dr. Edward Jenner.

PRESUMING that you are conversant with the practice of vaccine inoculation according to the instructions which I have formerly published, and that you may have seen, in addition to my general observations, those which I have since made and promulgated, respecting the "Varieties and Modifications of the Vaccine Pustule, occasioned by an herpetic and other eruptive states of the skin," I take the liberty of requesting to be informed whether the observations acquired in your own practice coincide with mine? That is to say, whether the vaccine vesicles, under these contingent circumstances, go through their course with the same regularity as when the skin is free from diseases of this description?

Secondly, Whether, on the other hand, such individuals are more liable to resist the legitimate action of vaccine lymph, when inserted into the arms, than those who are free from such eruptive affections?

Thirdly, Whether you have met with cases of small-pox, or what has been termed the varioloid disease, after vaccination: and, if so, whether in such cases you ascertained those deviations at the time of vaccination in the progress of the pustules on the arms, which I have described as liable to take place when the skin is affected with herpetic and other eruptions?

As you may not have the paper before you to which I here allude, nor the short series which followed it, I will point out the periods of their publication, and where they are to be found. The first was published in the Medical and Physical Journal, No. 66, for August 1804, and gives an outline of the subject, of some extent. It points out the fact, that a single serous blotch upon the skin, existing during the progress of the vaccine vesicles on the arms, may occasion such irregularity and deviation from correctness, that vaccination, under such circumstances, cannot be perfectly depended on.

I have found abrasions of the cuticle to produce the same effect; such, for example, as we find in the nurseries of the opulent as well as the cottages of the poor, behind the ears, and upon many other parts where the cuticle is thin. Happily we find no irregularity in the vaccine vesicle in an uncontaminated skin; but we find it if the skin is beset with these herpetic blotches, or even simple serous oozings from an abraded cuticle. It is not to be considered as of less consequence when occupying a small space; a speck behind the ear, which might be covered by a split-pea, being capable of disordering the progress of the vaccine vesicle. Dandriffe may be considered as a malady of this class, the incrustation on the scalp being formed from excoriation beneath; and however slight, for there is every gradation between a thin scurfy layer of a dirt-looking substance, or even patches of this thin crust, and tinea itself. However, fortunately for the safety of the vaccine practice, and fortunately, too, for the ease of the practitioner, all these affections of the skin may be removed with very little trouble.* Sore eyelids are also impediments to constitutional vaccination.

The second paper relating to this subject was given by the late Dr. Willan, in answering to the following interrogatory, addressed to me by himself:† "What are the changes produced in the vesicle, when a person is affected during vaccination with the shingles, the vesicular ringworm, or impetigo?"

To this question I made a full, and, I believe, a satisfactory reply. Its purport will be shown by quoting a few sentences from it. "To answer this question in its fullest extent would lead me through a wide field of observation, which I mean to go over at a future time; but the following answer may probably convey to you as much information upon the subject as you may now require." "Vaccination, under the circumstances you mention, usually produces a striking deviation from the perfect character of the vaccine vesicle at some period or other of its progress, but more frequently in its early than in its declining stages; indeed, it is commonly perceptible in a day or two after inoculation. It would be difficult, perhaps impossible, without the aid of drawings, to give a correct description of the varieties which an herpetic state of the skin is capable of producing, from those trifling deviations which prove no impediment to the vaccine security, up to that point of imperfection in the vesicle which affords no security at all. Perhaps I commit an error in saying "no security at all;" for it strikes me that the constitution loses its susceptibility of small-pox contagion, and its capability of producing the disease in its perfect and ordinary state, in proportion to the degree of perfection which the vaccine vesicle has put on in its progress, and that the small-pox, taken subsequently, is modified accordingly.‡ When no deviation takes place in the ordinary course of the vaccine vesicles, or when it is inconsiderable, the herpetic blotches or vesicles, of whatever kind they may be, often assume (sometimes as early as the third or fourth day after the insertion of the vaccine fluid,) a new character, not unlike the vaccine,

* The most effectual application which I know for subduing these cuticular diseases that produce impediment, is the unguentum hydrargyri nitratis, as much lowered with unguentum cetacei, or any other bland ointment, as the irritability of the subject may require. The dandriffe demands a double process: the first consists in removing the incrustation, the second in subduing the oozing. There are skins that will not bear unctuous applications: the desiccative lotions may then be made use of two or three times a-day; such as those prepared with the sulphate of zinc or superacetate of lead, &c.

† It was published in the year 1806, in his Treatise on Vaccine Inoculation.

‡ Further observation has confirmed this opinion, and has also developed much other curious matter respecting the spontaneous blending of the herpetic with the vaccine fluid, through the medium of the constitution, when under the influence of herpes.

and, keeping pace in their progress with the vesicles on the arm, die away with them, leaving the skin smooth."

These two papers comprehend, first, the simple fact of important deviations being produced by diseases in pre-occupation of the skin; and, secondly, a general account of the characters of these deviations, and their differing degrees of influence upon the vaccine protection.

Some further observations were published by Dr. Wilson Philip, of Worcester, in an Appendix to his work on Febrile Diseases, who requested some information from me on this interesting subject. This letter goes more into detail than the former, though its purport is the same,—namely, to guard the practitioner against the insidious influence of a diseased skin, when he vaccinates. It will be an object of future consideration to enter more generally into the minutiæ of this subject; but a sketch like this does not afford scope for the completion of such a design. Let me advise every practitioner not to confine his cautions, nor to narrow my meaning, to one class of eruptive affections. In short, every disease of the skin which may be called *serous*, or one that sends out a fluid capable of conversion into a scab, has the power of exerting this modifying and counteracting influence; and I have also seen purulent fluids exert a similar influence in producing deviations. If I was asked what were the other actual impediments to perfect vaccination, as a general answer I should say, that I scarcely know any other except spurious matter,* or impediments too obvious to require my naming them here, such as deranging the vaccine vesicle in its progress, by incautiously robbing it of its contents, or producing a new action by external violence.

*Berkeley; Feb.* 1821.

---

*Case of Hydrophobia.* By William Mines, Surgeon.

ROBERT MERRY, footman to Robert Sheriffe, Esq. of Diss, applied to me for medical aid, December 1st, 1820, eight o'clock, A.M. complaining of having had a very restless night: he could get no sleep, from unpleasant symptoms about his throat, with extreme difficulty of swallowing even his saliva; and at times his breathing was much oppressed. His pulse was under

* I am happy to see that these interruptions are now discovered in Germany; as appears in Professor Hufeland's Journal for June 1819, an extract of which is given in the London Medical Repository, vol. xiv. p. 502.

In addition, see Bateman's "Synopsis of Cutaneous Diseases," pp. 222, 223; Cross's "History of the Variolous Epidemic at Norwich, 1820," pp. 60 *et seq.* 196 and 288. I was lately puzzled to find the cause of irregularity in a vaccine vesicle, the skin being free from any apparent eruption: upon minute inquiry, I discovered a whitlow on the thumb, in which suppuration had taken place.

